# Uninephrectomy in Rats on a Fixed Food Intake Potentiates Both Anorexia and Circulating Cytokine Subsets in Response to LPS

**DOI:** 10.3389/fimmu.2015.00641

**Published:** 2015-12-22

**Authors:** Denis Arsenijevic, Jean-Pierre Montani

**Affiliations:** ^1^Division of Physiology, Department of Medicine, University of Fribourg, Fribourg, Switzerland; ^2^National Center of Competence in Research Kidney Control of Homeostasis (Kidney.CH), Zurich, Switzerland

**Keywords:** uninephrectomy, LPS, brain, cytokines, neuropeptides, anorexia, rats, Sprague-Dawley

## Abstract

Recent human studies have suggested that mild reduction in kidney function can alter immune response and increase susceptibility to infection. The role of mild reduction in kidney function in altering susceptibility to bacterial lipopolysaccharide (LPS) responses was investigated in uninephrectomized rats compared to Sham-operated controls rats 4 weeks after surgery. Throughout the 4 weeks, all rats were maintained under mild food restriction at 90% of *ad libitum* intake to ensure the same caloric intake in both groups. In comparison to Sham, uninephrectomy (UniNX) potentiated LPS-induced anorexia by 2.1-fold. The circulating anorexigenic cytokines granulocyte-macrophage colony stimulating factor, interferon-γ, tumor necrosis factor-α, and complement-derived acylation-stimulating protein were elevated after LPS in UniNX animals compared to Sham animals. Interleukin(IL)1β and IL6 pro-inflammatory cytokines were transiently increased. Anti-inflammatory cytokines IL4 and IL10 did not differ or had a tendency to be lower in UniNX group compared to Sham animals. LPS-induced anorexia was associated with increased anorexigenic neuropeptides mRNA for pro-opiomelanocortin, corticotrophin-releasing factor, and cocaine–amphetamine-regulated transcript in the hypothalamus of both Sham and UniNX groups, but at higher levels in the UniNX group. Melanocortin-4-receptor mRNA was markedly increased in the UniNX group, which may have contributed to the enhanced anorexic response to LPS of the UniNX group. In summary, UniNX potentiates pro-inflammatory cytokine production, anorexia, and selected hypothalamic anorexigenic neuropeptides in response to LPS.

## Introduction

Chronic kidney disease is associated with an activation of the immune system and a chronic inflammatory state (1). Studies in kidney-transplant recipients have shown that there is a specificity of infections associated with reduced kidney function (2–4), but there is the confounding effect of immunosuppressive therapy. Thus, the situation concerning mild reduction in kidney function and infection is less clear. A recent study in humans has suggested that mild or moderate chronic kidney disease increases the risk of postoperative infections (5). There are also data showing that mild reduction in kidney function in elderly persons is associated with increased circulating cytokines that are biomarkers for inflammation (6).

Infection activates host immune cells to produce cytokines. Susceptibility to acute bacterial Gram-negative infection can be mimicked by lipopolysaccharide (LPS) injection. LPS is used to induce pro-inflammatory cytokine production by immune cells. These cytokines are believed to be responsible in inducing pathology and metabolic alterations in LPS-injected mice (7). There is evidence that in humans, reduced kidney function augments immune cell cytokine production to LPS (8). LPS can induce anorexia when injected in the periphery (intraperitoneally) with several pro-inflammatory cytokines mediating this anorexia (9). However, cytokines injected directly into the brain (intracerebroventricularly) can also induce anorexia (10). It is generally believed that peripheral LPS induces peripheral cytokines, which then act on the brain on neurons that are involved in food intake regulation. We have previously shown that increased basal circulating cytokines (11) during a chronic infection results in excessive responses to LPS resulting in enhanced anorexia and hyperinduction of certain circulating cytokines. LPS/cytokines induce anorexia by acting on hypothalamic neural feeding pathways (10, 12, 13). Hypothalamic anorexigenic neuropeptides involved in feeding are involved in LPS anorexia. These include pro-opiomelanocortin (POMC) (14), cocaine- and amphetamine-regulated transcript (CART) (15), corticotrophin-releasing factor (CRF) (16), and melanocortin-4-receptor (MC4R) (17, 18).

To test the hypothesis that mild reduction in kidney function increases the inflammatory and anorexic responses to LPS, we analyzed in uninephrectomized rats, compared to Sham-operated rats, the response to LPS on peripheral cytokines, on food intake, and on selected hypothalamic anorexigenic neuropeptides involved in food intake regulation. We used uninephrectomy (UniNX) because it is a model of mild reduction in kidney function (19) in contrast to other renal dysfunction models, such as 5/6 nephrectomy (20), which result generally in a greater degree of renal impairment with problems related to uremia and accumulation of toxic compounds.

## Materials and Methods

### Animal Preparation and Experimental Protocol

#### Animals and Diets

All experimental protocols were approved by the Ethical Committee of the Veterinary Office of Fribourg, Switzerland. Male Sprague-Dawley rats were purchased from Elevage Janvier (Le Genest-St-Isle, France). Rats arrived at 5 weeks of age with an average weight of 160 g/animal. They were placed in individual cages and given pellet food and water *ad libitum*. After a week acclimation period, rats were either Sham operated or uninephrectomized (UniNX) by removal of the left kidney. After surgery, animals were put under a fixed food intake (90% of *ad lib-*fed diet) diet of normal chow paste. Dry food powder (Normal chow diet: Cat. No. 3433, Provimi-Kliba, Cossonay, Switzerland) was mixed with an equal amount of tap water, which was prepared daily (90 kcal/rat) and given in food cups (9.00 a.m.–11.00 a.m.). We chose to put the rats under a fixed food intake (90% of *ad lib*-fed diet) to ensure the same caloric intake between Sham and UniNX rats. *Ad lib* feeding results in uncontrolled levels of nutrition, which can influence metabolites, hormones, inflammation, oxidative stress, and parameters of interest (21). Rats were kept in individual cages and had free access to water. The environmental temperature was maintained at 22 ± 1°C in a room with a 12 h light/dark cycle (light 7.00 a.m.–7.00 p.m.).

#### Surgery

Left UniNX and Sham surgery were performed as previously described (19).

#### Lipopolysaccharide Experimental Protocol

**Figure 1:**
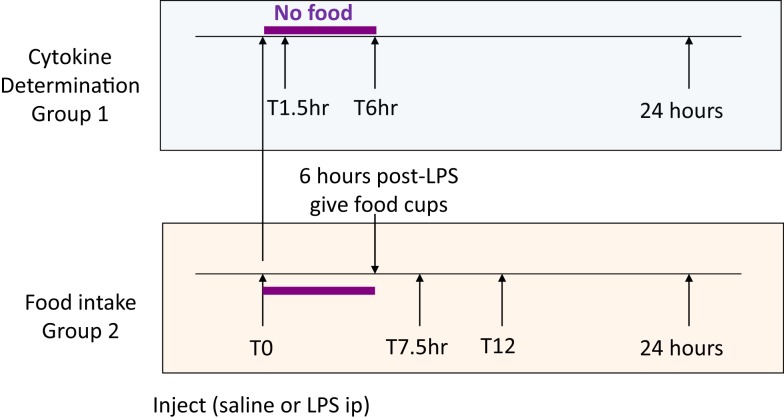
**Time lines for cytokine (group 1) and food intake (group 2) measurements**.

Four weeks after surgery, Sham-operated (body weight 463 ± 13 g) and UniNX rats (461 ± 13 g) were randomly separated into groups (*n* = 8 per group). Four groups of Sham-operated rats and four groups of UniNX rats were sacrificed for cytokine determination before and at 1.5, 6, and 24 h after intraperitoneal injection of 100 μg/kg LPS (*E. coli* 0111:B4, Sigma, Buchs, Switzerland) around 7 a.m., time T0 (Figure 1, group 1). Two additional groups (Sham and UniNX) were injected first intra-peritoneally with pyrogen free saline and 4 days later with LPS for food intake determination (Figure 1, group 2).

#### Food Intake Determination

Food was given to the rats only 6 h following the injection in order to allow sufficient time for the effects of LPS on food intake to develop (22). Food intake was then measured at 7.5, 12, and 24 h after saline or LPS injection.

### Blood and Brain Collection

We collected serum and plasma (in EDTA or heparin-coated tubes) on ice and centrifuged at 4°C at 3,000 rpm in a microcentrifuge. Serum and plasma were then kept at −20°C until analyzed. The hypothalamic regions were cut out from the brain and then snap frozen in liquid nitrogen and stored at −80°C until ready for use in analysis. Tissues were pulverized in liquid nitrogen prior to analysis.

### Circulating Cytokines

ELISA kits were purchased from eBioscience (San Diego, CA 92121, USA) for the following cytokines: interleukin-4, IL6, IL10, tumor necrosis factor alpha (TNFα), interferon-gamma (IFNγ), and granulocyte macrophage colony stimulating factor (GM-CSF). IL1β ELISA kit was purchased from R&D systems, Abingdon, UK. Acylation-stimulating protein (ASP) was measured by ELISA from MyBiosource, San Diego, CA, USA.

### RT-PCR in Brain Hypothalamus

After sacrificing, brain hypothalamic regions were cut out and placed in liquid nitrogen and then stored at −80°C. Total RNA was isolated as previously described (23). The RNA was then treated with DNase, after which it was reverse transcribed (Promega). Thereafter, we ran a RT-PCR. Samples were incubated in the iCycler instrument (BioRad, iCycler iQ™, Version 3.1.7050) for an initial denaturation at 95°C for 3 min, followed by 40 cycles of amplification. Each cycle consisted of 95°C for 10 s, 60 or 62°C for 45 s, and finally 95, 55, and 95°C for 1 min each. SybrGreen fluorescence emission was determined after each cycle. The relative amount of each mRNA was quantified using the iCycler software. Amplification of specific transcripts was confirmed by melting curve profiles generated at the end of each run. Cyclophilin was used as the control for each study and the relative quantification for a given gene was normalized to cyclophilin mRNA values. For a complete list of primers and their sources, see Table 1.

**Table 1:** **RT–PCR primers**.

Primer(source reference)	Sense 5′–3′	Anti-sense 5′–3′
GM-CSFR*a* (24)	GCT GCA CCC GAT GAC ATC	GAA GGC GAA GGC GTT GTC
IFNGR*a* (25)	TTT GGA TGC TGC TTG TTG CTC CTC	AGT TCT TCC TGC TCT CTG GTGCTT CT
C5L2 (26)	TTG CAG TGG CTT CTT CAC AC	GAT ACC TTG GTC ACG CAC CT
CART (27)	AGA AGA AGT ACG GCC AAG TCC	CAC ACA GCT TCC CGA TCC
POMC (27)	CCT GTG AAG GTG TAC CCC AAT GTC	CAC GTT CTTGAT GGC GTT C
MC4R (27)	TAT GGT ACT GGA GCG CGT AA	TCA GAC GGA GGA TGC TAT GA
CRF (28)	CTC TCT GGA TCT CAC CTT CCA C	CTA AAT GCA GAA TCG TTT TGG C
Cyclophilin (29)	TCA GGG CTC TTG AAG TCC C	CAG AAA ATC ACA GCA GCC ACC

### Statistical Analysis

Data are presented as means ± SE. Statistical analysis was performed using ANOVA Tukey–Kramer multiple comparison test. A value of *p* < 0.05 was considered as significant.

## Results

### Food Intake

During the light phase at 7.5 and 12 h, food intake tended to be higher in UniNX-saline rats than in Sham-saline rats, but the difference was not statistically significant. Food intake in Sham-LPS and UniNX-LPS at these two time points were significantly reduced (*p* < 0.001) compared to their saline counterparts. No differences were observed between Sham-LPS and UniNX-LPS groups at the two time points. The majority of food intake for both Sham and UniNX occurred in the dark phase after 12 h.

**Figure 2:**
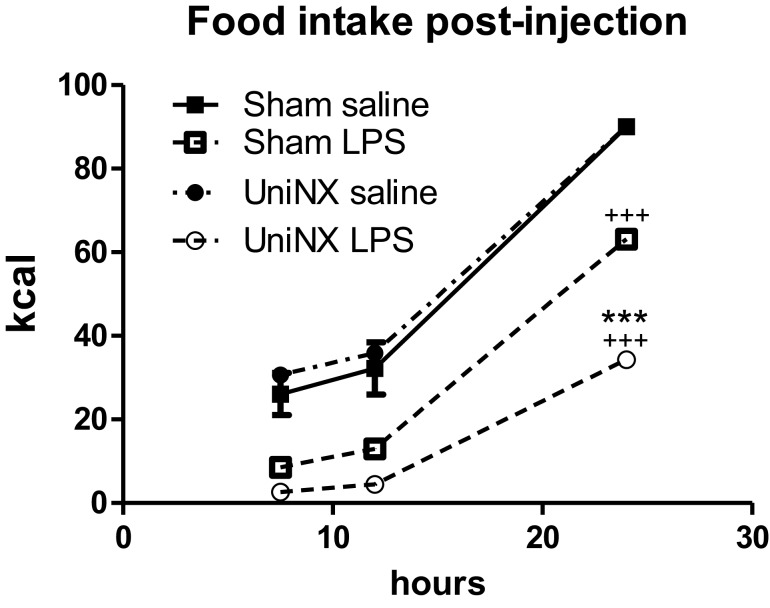
**Cumulative food intake over 24 h for Sham and UniNX rats treated with i.p. saline or LPS (100 μg/kg body weight)**. Values are means ± SE; *n* = 8/group. ^+++^*p* < 0.001 corresponds to Sham vs. Sham-LPS or UniNX vs. UniNX-LPS. ***p < 0.001 corresponds to Sham-LPS vs. UniNX-LPS.

Cumulative food intake over 24 h in saline-Sham and saline-UniNX rats was identical since both groups consumed 100% of their allotment of the isocaloric diet (Figure 2). LPS administration led to anorexia in both Sham and UniNX animals. LPS induced a 29% reduction in food intake in Sham animals relative to the Sham-saline control group (Figure 2). In UniNX, LPS induced a 62% reduction in food intake relative to the UniNX-saline control group (Figure 2). Thus, by the end of the 24-h period following LPS administration, UniNX had potentiated LPS anorexia 2.1-fold, relative to the Sham-LPS group (Figure 2).

### Circulating Cytokines

**Figure 3:**
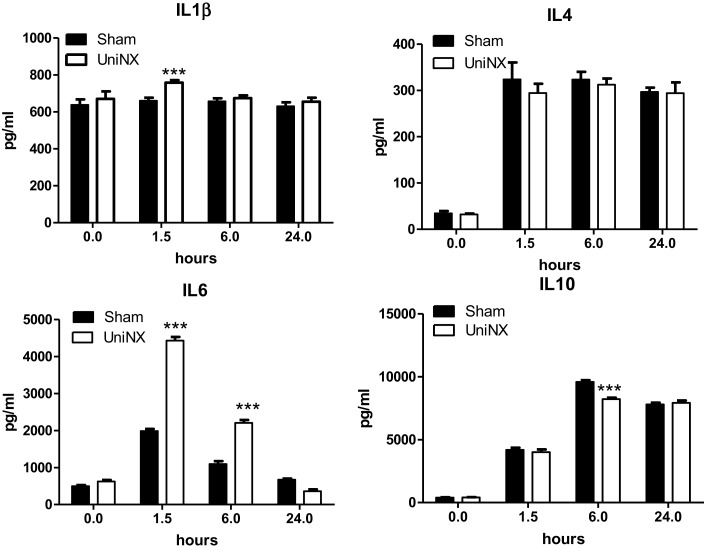
**Circulating levels of IL1β, IL4, IL6, and IL10, in Sham-operated and UniNX rats at 0, 1.5, 6, and 24 h after i.p. LPS injection**. Values are means ± SE; *n* = 8/group. ***p < 0.001 corresponds to Sham vs. UniNX.

**Figure 4:**
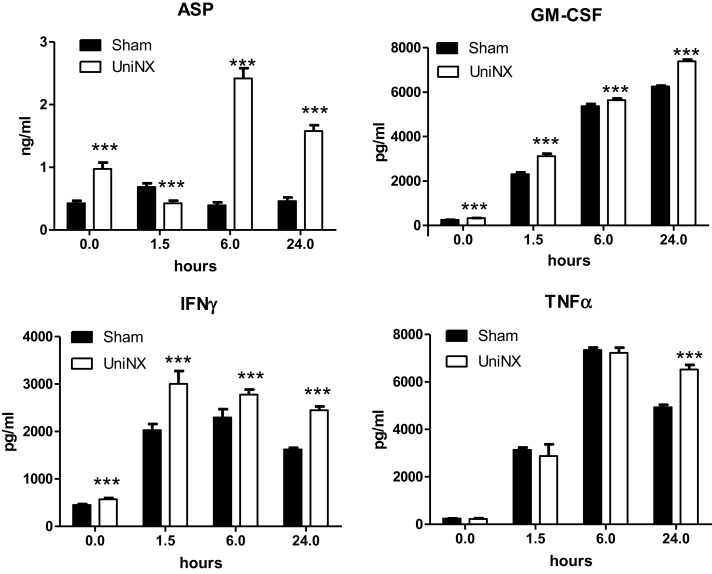
**Circulating levels of ASP, GM-CSF, IFNγ, and TNFα, in Sham-operated and UniNX rats 1.5, 6, and 24 h after i.p. LPS injection**. Values are means ± SE; *n* = 8/group. ***p < 0.001 corresponds to Sham vs. UniNX.

The pro-inflammatory cytokines IL1β and IL6 showed a transient increase in UniNX group after LPS compared to Sham-LPS group (Figure 3). In contrast, anti-inflammatory cytokines IL4 and IL10 were stimulated and sustained up to 24 h, although the response tended to be lower in the UniNX group (Figure 3). Basal circulating levels of ASP, GM-CSF, and IFNγ were higher in the UniNX animals relative to the Sham group (Figure 4). Each of these three anorexic cytokines and TNFα exhibited sustained increases for the 24-h period following the LPS injection, with the levels being significantly greater in UniNX animals relative to the LPS-treated Sham group (*p* < 0.001) (Figure 4).

### Hypothalamic Acylation-Stimulating Factor-Receptor (C5L2), GM-CSF- Receptor, and IFNγ-Receptor mRNA Levels

**Figure 5:**
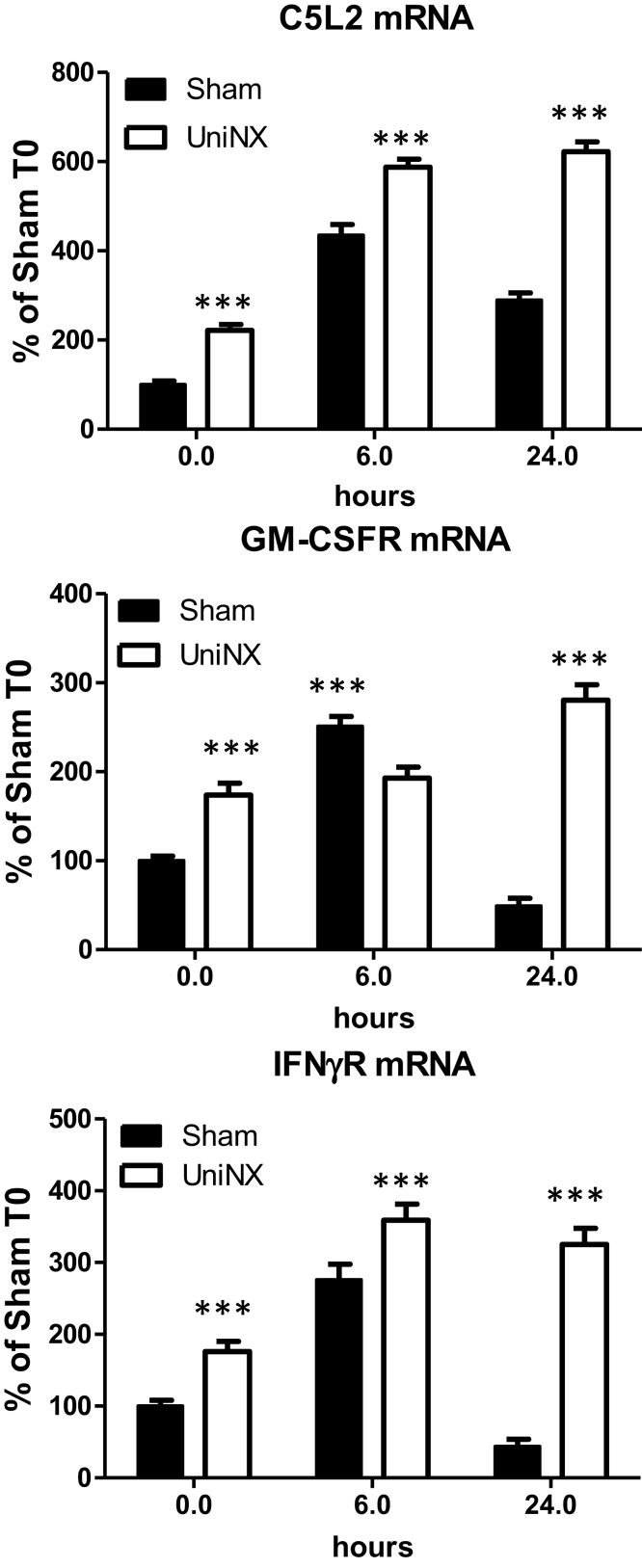
**Hypothalamic mRNA levels of receptors ASP (C5L2), GM-CSFR, and IFNγR in Sham and UniNX animals after 6 and 24 h after i.p. LPS injection**. Values are means ± SE; *n* = 8/group. ***p < 0.001 corresponds to Sham vs. UniNX.

Basal levels of hypothalamic C5L2, IFNγR, and GM-CSFR mRNA were significantly higher in UniNX than in Sham animals (Figure 5). Following LPS, the mRNA levels of these receptors remained significantly higher in UniNX than in the Sham group (Figure 5) with one exception (GM-CSFR at 6 h).

### Hypothalamic Neuropeptides mRNA

**Figure 6:**
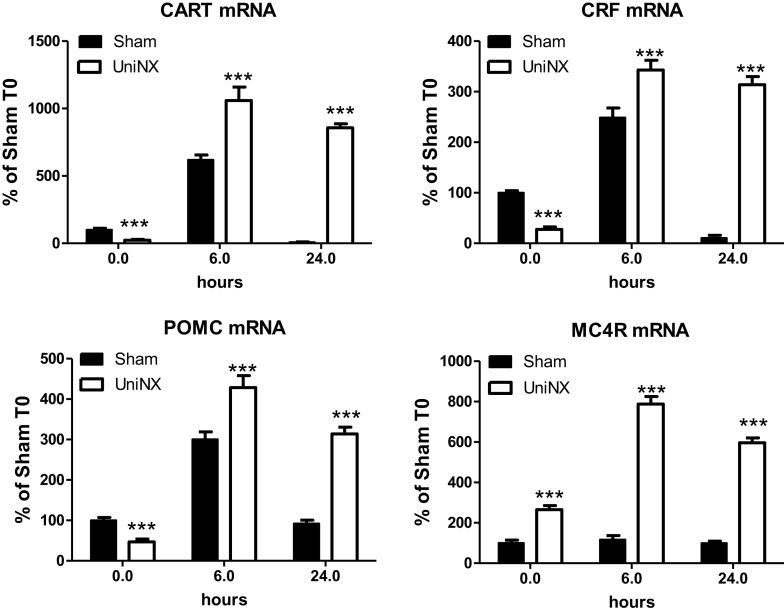
**Hypothalamic mRNA levels of anorexigenic neuropeptides CART, CRF, MC4R, and POMC in Sham and UniNX animals after 6 and 24 h after i.p. LPS injection**. Values are means ± SE; *n* = 8/group. ***p < 0.001 corresponds to Sham vs. UniNX.

UniNX decreased the basal level of certain hypothalamic neuropeptide mRNAs. CART, CRF, and POMC mRNA levels were lower compared to Sham levels (Figure 6). In contrast, the level of MC4R mRNA was increased by UniNX (Figure 6). LPS-potentiated increases in all selected neuropeptides and MC4R measured in UniNX animals compared to Sham counterparts (Figure 6).

In general, LPS-induced higher levels of anorexigenic neuropeptides CART, CRF, and POMC (Figure 6). Furthermore, UniNX showed higher levels of these neuropeptides, which peaked at 6 h post-LPS (Figure 6). Twenty-four hour post-LPS, these neuropeptides remained elevated in UniNX animals whereas they returned to basal levels in the Sham animals (Figure 6).

## Discussion

The major findings of our study are that UniNX potentiates both cytokine production and anorexia induced by LPS. In our previous study (19), we showed that UniNX led after 4 weeks to a 46% increase in plasma urea and a 47% increase in plasma cystatin C in comparison to Sham-operated controls. This corresponds to a 32% decrease in glomerular filtration rate (GFR), assuming a hyperbolic relationship between GFR and plasma cystatin C. We are thus studying a model of mild reduction of kidney function, in contrast to experimental models of more severe renal impairment.

Four weeks post-UniNX, basal levels of a subset of circulating cytokines, including IFNγ, GM-CSF, and the complement-derived ASP, were mildly elevated in the UniNX group compared to Sham animals as previously described (19). The low-level increases in basal IFNγ, GM-CSF, and ASP, however, did not affect total food intake over 24 h (fixed to 90% of *ad libitum* intake) in the UniNX animals compared to the Sham animals before LPS. The pro-inflammatory cytokines TNFα, GM-CSF, IFNγ, and ASP were induced to higher levels by LPS in UniNX animals over 24 h compared to the Sham group. TNFα and IFNγ have been shown to play an important role in LPS-induced anorexia (9, 30). Circulating ASP increases have been observed in kidney disease in humans (31–33) and can reduce body weight (34). Cytokines act on the brain to induce anorexia. This effect is believed to be mediated by an indirect action on blood vessels and the induction of cytokine production in the brain. Alternatively, they can act by gaining access to the brain and directly act on brain cells with respective cytokine receptors. Although these mechanisms have been described for TNFα (9, 35), IL1β (36, 37), IL6 (38), and their receptors (9, 39–43), the pathways for IFNγ, GM-CSF, and ASP in the context of LPS anorexia, however, have not been established. Here we show that mRNAs for these receptors – ASP (C5L2), GM-CSFR, and IFNγR are expressed in the hypothalamus suggesting that the brain can respond to these three immune-mediators. Previous studies have shown that injection of ASP, GM-CSF, and IFNγ within the cerebral ventricles resulted in anorexia (34, 44, 45). LPS induces the three receptors to higher levels in the hypothalamus of UniNX animals compared to Sham group, suggesting that sensitivity to these cytokines may be altered in the brain. Other circulating pro-inflammatory cytokines in the UniNX group may play a role such as IL1β and IL6, even though they peak early and then return to basal or Sham levels by 24 h. Interestingly, anti-inflammatory cytokines such as IL4 and IL10 had a tendency to be lower in the UniNX group in response to LPS. This may suggest that increased cytokine levels in the UniNX group may not be only the result of reduced cytokine clearance by the kidney. Reduced IL10 levels have also been reported in human kidney disease (46). Our data are consistent with the idea that reduced kidney function alters cytokines subsets and that cytokines are involved in pathology after nephrectomy (47). Potentiation of cytokine production by LPS also occurs in other models of disease including infection/inflammation (48, 49) and in human kidney disease (8). Neuropeptide potentiation has been shown to occur in anorexia nervosa (50) and bulimia nervosa (51).

Basal levels of anorexigenic neuropeptides CART, CRF, and POMC were decreased in UniNX animals compared to Sham-operated controls, whereas MC4R was increased. These observations are consistent with the fact that food intake data at 7.5 and 12 h post-saline in UniNX animals had a tendency to be a little bit higher. Higher circulating anorexic cytokines levels were observed after LPS in UniNX compared to Sham animals. This was associated with a more than twofold potentiation of the anorexic response to LPS in UniNX animals, relative to the Sham group. This differential increased anorexia in UniNX animals compared to Sham animals may be explained by increases in anorexigenic hypothalamic neuropeptides POMC, CRF, and CART mRNA levels. Our study is in agreement with other studies that LPS induces increases hypothalamic mRNA for POMC (52), CART (53, 54), and CRF (16, 52). Furthermore, i.c.v. injection of ASP has been shown to increase hypothalamic POMC mRNA (34). In rodent kidney disease, it has been shown that MC4R and POMC play a role in anorexia, modifying body composition, and this involves cytokines (55, 56). Our study suggests a role for mild reduction of kidney function in altering the basal levels of circulating cytokines and mRNA of brain neuropeptides (MC4R, POMC). Furthermore, basal hypothalamic neuropeptides levels have also been shown to be altered following UniNX. The consequence of these changes for body composition (19) requires additional investigation. Further attention is also required to determine why in the UniNX group LPS potentiates the transcriptional mRNA expression levels of all the measured neuropeptides, but not in Sham animals.

In summary, we show in young male rats that UniNX, a model of mild reduced kidney function, potentiates pro-inflammatory cytokine production, anorexia, and selected hypothalamic anorexigenic neuropeptides in response to LPS. This suggests that UniNX could alter susceptibility to infections. UniNX also alters basal inflammation, which involves a subset of circulating cytokines. These changes in cytokines could have several consequences on the host, beneficial or detrimental depending on the infection or the inflammatory process. This may also explain why there may be a specific profile of infections associated with kidney disease (57). Further evidence of altered immune response in patients with kidney disease may explain problems encountered with vaccination of patients (58). Therefore, any situation that depends on an immune response could have an impact on the host due to reduced kidney function.

## Author Contributions

Conceived and designed the experiments: DA, J-PM. Performed the experiments: DA. Analyzed the data: DA, J-PM. Wrote paper: DA, J-PM. Edited manuscript: DA, J-PM.

## Conflict of Interest Statement

The authors declare that the research was conducted in the absence of any commercial or financial relationships that could be construed as a potential conflict of interest. The Reviewer Daisuke Kamimura and the Handling Editor, Masaaki Murakami declare their shared affiliation and the Handling Editor states that the review process nevertheless met the standards of a fair and objective review.

## Abbreviations

ASP, acetylation-stimulating factor; C5L2, ASP receptor; CART, cocaine–amphetamine-regulated transcript; CRF, corticotrophin-releasing factor; GM-CSF, granulocyte-macrophage colony-stimulating factor; IFNγ, interferon-gamma; IL, interleukin; LPS, lipopolysaccharide; POMC, pro-opiomelanocortin; MC4R, melanocortin-4-receptor; UniNX, uninephrectomy.
